# Improving Protein Gamma-Turn Prediction Using Inception Capsule Networks

**DOI:** 10.1038/s41598-018-34114-2

**Published:** 2018-10-24

**Authors:** Chao Fang, Yi Shang, Dong Xu

**Affiliations:** 1 Department of Electrical Engineering and Computer Science, University of Missouri, Columbia, Missouri 65211 USA; 2 Christopher S. Bond Life Sciences Center, University of Missouri, Columbia, Missouri 65211 USA

## Abstract

Protein gamma-turn prediction is useful in protein function studies and experimental design. Several methods for gamma-turn prediction have been developed, but the results were unsatisfactory with Matthew correlation coefficients (MCC) around 0.2–0.4. Hence, it is worthwhile exploring new methods for the prediction. A cutting-edge deep neural network, named Capsule Network (CapsuleNet), provides a new opportunity for gamma-turn prediction. Even when the number of input samples is relatively small, the capsules from CapsuleNet are effective to extract high-level features for classification tasks. Here, we propose a deep inception capsule network for gamma-turn prediction. Its performance on the gamma-turn benchmark GT320 achieved an MCC of 0.45, which significantly outperformed the previous best method with an MCC of 0.38. This is the first gamma-turn prediction method utilizing deep neural networks. Also, to our knowledge, it is the first published bioinformatics application utilizing capsule network, which will provide a useful example for the community. Executable and source code can be download at http://dslsrv8.cs.missouri.edu/~cf797/MUFoldGammaTurn/download.html.

## Introduction

Protein tertiary structure prediction has been an active research topic since half a century ago^1–3^. Because it is challenging to directly predict the protein tertiary structure from a sequence, it has been divided into some sub-problems, such as protein secondary and super-secondary structure predictions. Protein secondary structures consist of three elements such as alpha-helix, beta-sheet and coil^4^. The coils can be classified into tight turns, bulges and random coil structures^5^. Tight turns can be further classified into alpha-, gamma-, delta-, pi- and beta -turns based on the number of amino acids involved in forming the turns and their features^6^. The tight turns play an important role in forming super-secondary structures and global 3D structure folding.

**Figure 1:**
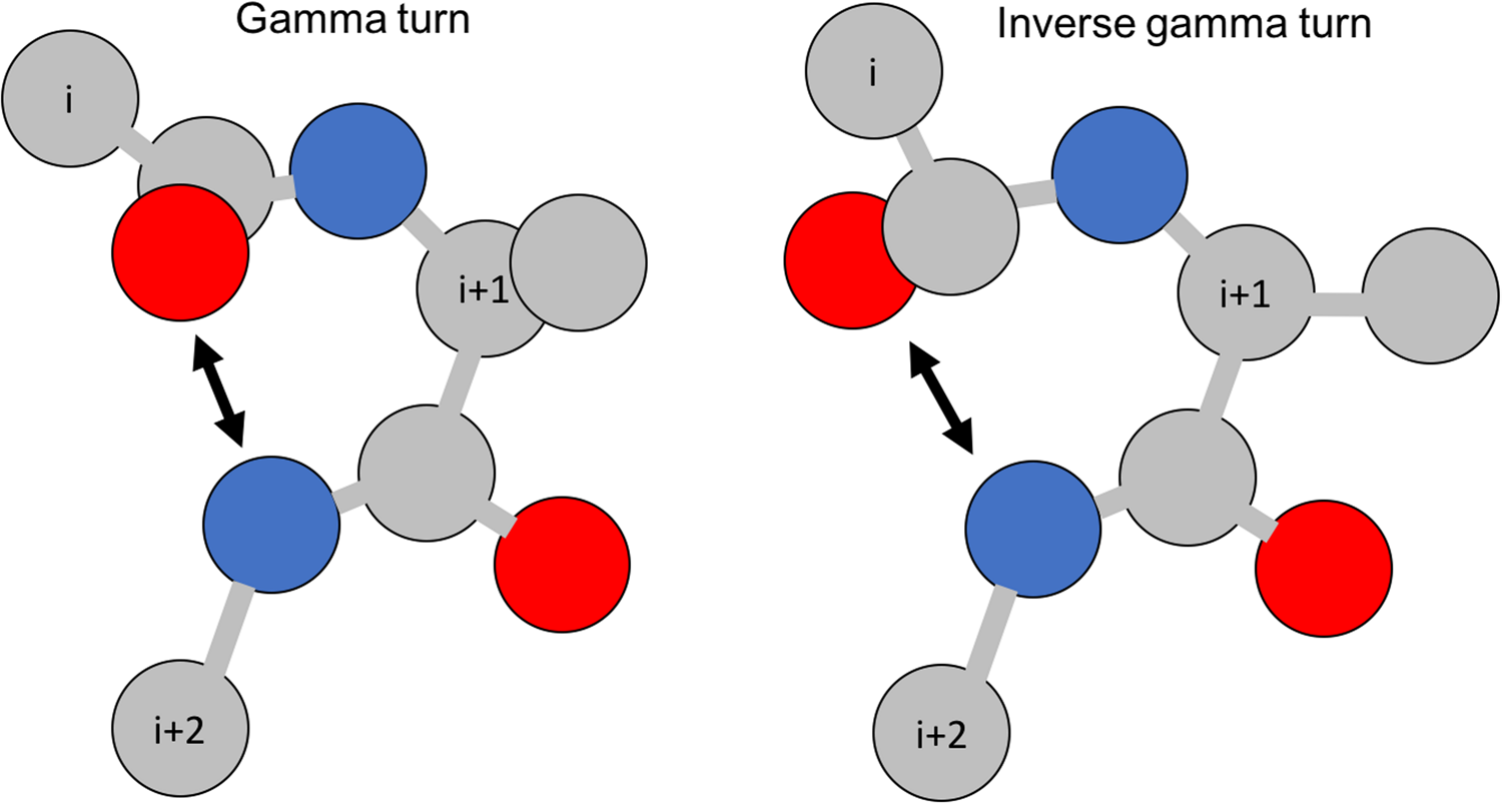
An illustration of gamma-turns. Red circles represent oxygen; grey circles represent carbon; and blue circles represent nitrogen.

Gamma-turns are the second most commonly found turns (after beta-turns) in proteins. By definition, a gamma-turn contains three consecutive residues (denoted by *i, i* + 1, *i* + 2) and a hydrogen bond between the backbone *CO*_*i*_ and the backbone *NH*_*i*+2_ (see Fig. 1). There are two types of gamma-turns: classic and inverse^7^. Gamma-turns account for 3.4% of total amino acids in proteins^8^. They can be assigned based on protein 3D structures by using PROMOTIF software^9^. There are two types of gamma-turn prediction problems: (1) gamma-turn/non-gamma-turn prediction^10–12^, and (2) gamma-turn type prediction^13–15^.

The previous methods can be roughly classified into two categories: statistical methods and machine-learning methods. Early predictors^10,11,16^ built statistical models and machine-learning methods to predict gamma-turns. For example, Garnier *et al*.^17^, Gibrat *et al*.^18^, and Chou^13^ applied statistical models while Pham *et al*.^12^ employed support vector machine (SVM). The gamma-turn prediction has improved gradually, and the improvement came from both methods and features used. Chou and Blinn^14^ applied a residue-coupled model and achieved prediction MCC 0.08. Kaur and Raghava^11^ used multiple sequence alignments as the feature input and achieved MCC 0.17. Hu and Li^19^ applied SVM and achieved MCC 0.18. Zhu *et al*.^20^ used shape string and position specific scoring matrix (PSSM) from PSIBLAST as inputs and achieved MCC 0.38, which had the best performance prior to this study. The machine-learning methods outperformed statistical methods greatly. However, the gamma-turns prediction performance is still low mainly due to two reasons: (1) gamma-turns are relatively rare in proteins, yielding a small training sample size; and (2) previous machine-learning methods have not fully exploited the relevant features of gamma-turns. The deep-learning framework may provide a more powerful approach for this problem than previous machine-learning techniques, like other deep-learning applications in protein sequence analysis and prediction^21–24^.

The recent deep neural networks have achieved outstanding performance in image recognition tasks, using methods such as inception networks^25^. The main components in the inception networks are inception blocks, each of which contains stacks of Convolutional Neural Networks (CNNs)^25^. To further capture the high-level relationships among features, Sabour *et al*.^26^ proposed a novel deep-learning architecture, named Capsule Network (CapsuleNet). The main components of CapsuleNet are capsules, which are groups of neuron vectors. The dimensions of a vector represent the characteristics of patterns, while the length (norm) of a vector represents the probability of existence. A CapsuleNet was trained for digit classification tasks^26^ and the length of a digit capsule represents the confidence of a certain digit being correctly classified and the dimensions of this digit capsule represent different features, such as the stroke thickness, skewness, and scale of a digit image.

Although CapsuleNets were primarily developed to capture orientation and relative position information of ‘entities’ or ‘objects’ in an image, in this paper we apply CapsuleNet to the biological sequence analysis problem from a different perspective. The motivation for applying CapsuleNet in gamma-turn prediction is due to its good properties: First, the dimension of a capsule can be used to reflect certain sequence properties of forming a gamma-turn. The capsule length also gives the confidence or prediction reliability of a predicted gamma-turn label. For example, the closer a capsule length (its norm value) is to 1, the more confident a predicted gamma-turn label is. Second, CapsuleNet contains capsules, each of which can detect a specific type of entity^26^. For an MNIST digit recognition task, each capsule was used to detect one class of digits, i.e. the first digit capsule detects 1’s; similarly, in this work, each capsule will be used to detect whether it is a classical turn, an inverse turn or non-turn. Also, compared to CNN, which has the invariance property, CapsuleNet has the equivariance property. The equivariance property means that a translation of input features results in an equivalent translation of outputs, which enables the network to generate features from different perspectives and hence requires a smaller sample size to train than previous CNN architectures. This is useful for many bioinformatics problems: even when the labelled data are scarce and limited, CapsuleNet can detect some high-level features and use them for robust classification. Third, the dynamic routing in CapsuleNet is similar to the attention mechanism^27^. The routing by agreement mechanism will let a lower-level capsule prefer to send its output to higher-level capsules whose activity vectors have a big scalar product with the prediction coming from the lower-level capsule. In other words, the capsules can “highlight” the most relevant features for a classification task, in this case, gamma-turn classification.

Here, we proposed a deep inception capsule network (DeepICN), which combines CapsuleNet with inception network for protein gamma-turn prediction. First, we performed extensive experiments to test the DeepICN performance under different conditions. Next, we show that the proposed network outperformed previous predictors utilizing traditional machine-learning methods such as SVM on public benchmarks. Last but not least, we further explored the features learnt by capsules and connected them back to the protein sequence to discover useful motifs that may form a gamma turn.

## Experimental Results

In this section, extensive experimental results of the proposed DeepICN with different hyper-parameters were tuned and tested using CullPDB and five-fold cross-validation results on GT320. The performance comparison with existing methods is presented.

### Experiment data set

1. CullPDB^28^ was downloaded on November 2, 2017. It originally contained 20,346 proteins with percentage cutoff 90% in sequence identity, resolution cutoff 2.0 Å, and R-factor cutoff 0.25. This dataset was preprocessed and cleaned up by satisfying all the following conditions: with length less than 700 amino acids; with valid PSIBLAST profile^29^ and HHblits profile^30^; with shape strings predicted by Frag1D^31^; and with gamma-turn labels retrieved by PROMOTIF^9^. After this, 19,651 proteins remained and CD-Hit^32^ with 30% sequence identity cutoff was applied on this dataset resulting in 10,007 proteins. We removed proteins with sequence identity more than 30% for an objective as most previous predictors did. This dataset was only used for experiments to explore deep neural network hyper-parameter tuning and DeepICN configurations. It was not used for comparison with previous predictors. For this dataset, a balanced dataset was built: positive gamma-turn labels were kept and an equal size of negative non-gamma-turn labels were selected to form a balanced dataset.
2. The benchmark **GT320**^8^ is a common data set used for benchmarking gamma-turn prediction methods. GT320 contains 320 non-homologous protein chains in total with 25% sequence identity cutoffs, and resolution better than 2.0 Å resolution. This benchmark was used to compare the performance with previous predictors. Each chain contains at least one gamma-turn. The gamma-turns were assigned by PROMOTIF^9^. Because all previous predictors applied and used five-fold cross-validation on this dataset, we did the same experiment as previous predictors for a fair comparison. It is worth mentioning that CullPDB was not used for training the model in this five-fold cross-validation experiment.

### Hyper-parameter tuning and model performance

Tables 1–4 show the exploration of DeepICN with different hyper-parameters. This set of experiments was to identify a better configuration of hyper-parameters for the deep networks using the CullPDB dataset. Since this network involves many hyper-parameters, only the major ones were explored. Table 1 shows how the sliding window size affects the model performance. In this experiment, 1000 proteins were randomly selected to form the training set, 500 for the validation set and 500 for the test set. Each experiment was performed with five times of data randomization.

**Table 1:** Effect of window size on MCC performance.

Window size	Test average MCC	Time (hr)	P-value on MCC
15	0.4458 (±0.0107)	0.18 (±0.11)	0.0115
17	0.4645 (±0.0062)	0.24 (±0.15)	—
19	0.4442 (±0.0049)	0.37 (±0.18)	0.0010
21	0.4548 (±0.0055)	0.43 (±0.20)	0.0499
23	0.4227 (±0.0076)	0.37 (±0.23)	0.0001
25	0.4369 (±0.0076)	0.45 (±0.25)	0.0005

**Table 2:** Effect of dropout on MCC performance.

Dropout	Train average MCC	Test average MCC	P-value on test MCC
No	0.9974 (±0.0015)	0.4439 (±0.0101)	0.1236
0.3	0.9857 (±0.0154)	0.4454 (±0.0049)	0.0843
0.4	0.9010 (±0.1457)	0.4515 (±0.0047)	0.4294
0.5	0.9377 (±0.0598)	0.4558 (±0.0092)	—
0.6	0.9159 (±0.0688)	0.4525 (±0.0111)	0.6647
0.7	0.8371 (±0.0920)	0.4604 (±0.0063)	0.4318
0.8	0.6072 (±0.1033)	0.4646 (±0.0249)	0.5228

**Table 3:** Effect of training size on training time and MCC performance.

Training size	Test average MCC	Time (hr)
500	0.4224 (±0.0035)	0.23 (±0.17)
1000	0.4553 (±0.0098)	0.87 (±0.03)
2000	0.4422 (±0.0204)	1.59 (±0.07)
3000	0.4752 (±0.0111)	2.38 (±0.09)
4000	0.4787 (±0.0147)	3.13 (±0.12)
5000	0.4717 (±0.0165)	3.91 (±0.14)

**Table 4:** Effect of dynamic routing on MCC performance.

Dynamic routing times	Test average MCC	Time (hr)	P-value on MCC
1	0.4454 (±0.0049)	0.44 (±0.16)	0.4644
2	0.4492 (±0.0086)	0.31 (±0.17)	—
3	0.4407 (±0.0032)	0.37 (±0.15)	0.1017
4	0.4497 (±0.0045)	0.32 (±0.18)	0.9276
5	0.4487 (±0.0061)	0.41 (±0.14)	0.9502

Table 1 shows how the sliding window size of the input affects the DeepICN performance. The larger the window size, the more training time it took for DeepICN. However, MCC may not grow as the window size increases. We chose the window size of 17 amino acids based on its peak MCC performance in the experiments. The t-test p-values show that the test MCC with a window size 17 compared to other window sizes is statistically significant.

Table 2 shows the dropout can effectively reduce the overfitting effects of DeepICN. If a dropout was not used, the network had very high over-fitting and the network cannot generalize well. The dropout rate 0.4–0.5 is reasonable as it is a compromise between the training and test prediction performance. We chose dropout 0.5 in our study. The p-value between the dropout of 0.5 and any of others was insignificant. Although the dropout of 0.8 had the highest test average MCC, its standard deviation (±0.0249) is also high, and hence, we did not use it.

Table 3 shows the effects of the training sample size on the DeepICN training speed and performance. More training data increased and training time and the model performance. However, after 3000 samples, the MCC performance did not improve significantly with more training data. This is consistent with the observation^26^ that CapsuleNet did not need a large dataset for training.

Table 4 shows the effect of number of dynamic routings on the performance. Dynamic routing is used in CapsuleNet similar to max-pooling in a CNN, but it is more effective than max-pooling in that it allows neurons in one layer to ignore all but the most active feature detector in a local pool in the previous layer. In this experiment, we fixed the other hyper-parameters searched in the above-mentioned experiments and studied how number of dynamic routing affected the performance. Considering the training time and the MCC performance, 2 routings are suitable, as more dynamic routings do not have significant improvement. The training time did not show large variations as the number of dynamic routings increases. This may be because our experiments used early stopping.

### Prediction confidence: the capsule length

**Figure 2:**
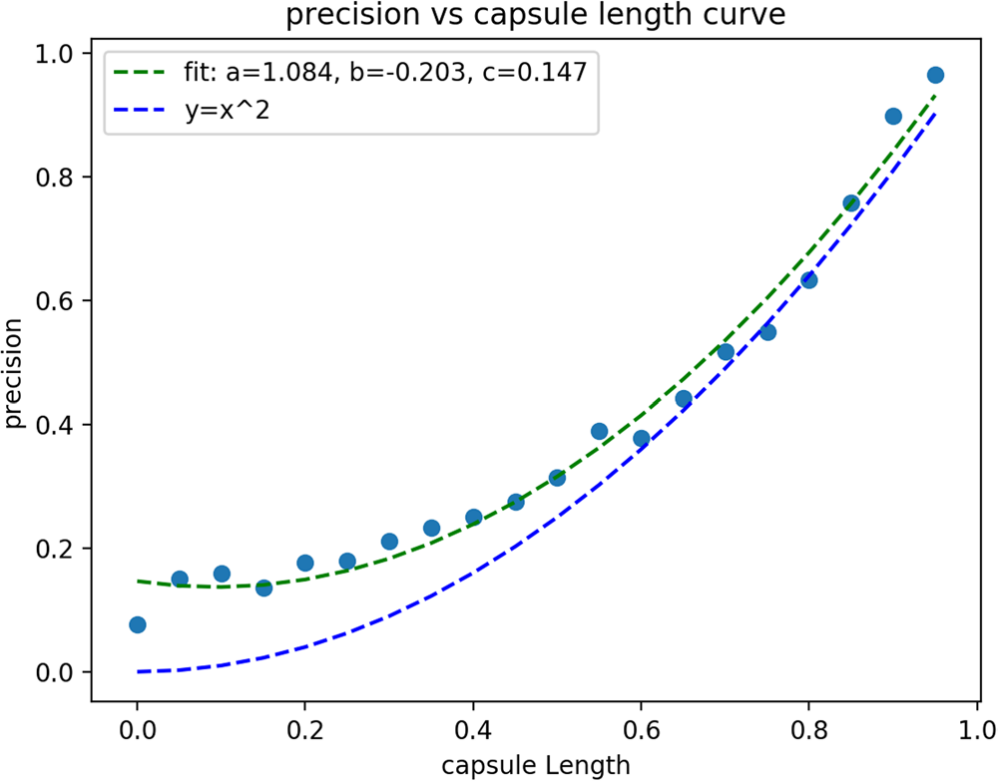
The fitting curve of precision (percentage of true positive in the bin) versus the capsule length. The green line is the fitting curve and the blue line (y = x^2^) is for reference.

Since the capsule length indicates the probability that the entity represented by the capsule is present in the current input^26^, the capsule length in the last layer can be used for prediction of gamma-turn and assessment of prediction confidence. The longer the turn capsule length is, the more confident the prediction of a turn capsule will be. Here, the capsule length in Turn Capsules can be used to show how confidence a gamma-turn is predicted. Specifically, a test set (with 5000 proteins containing 19,594 data samples) was fed into the trained DeepICN to get a capsule length vector. Then the capsule length vector that represents positive capsules were kept. Since all the capsule length values fall into the range between 0 and 1, they were grouped into bins with the width of 0.05, so that there are totally 20 bins. The precision of each bin can be calculated to represent the prediction confidence. Figure 2 shows the fitting curve of precision (percentage of correctly predicted gamma-turns, i.e., true positives in the bin) versus the capsule length. A nonlinear regression curve was used to fit all the points, yielding the following equation:

$$y=1.084{x}^{2}-0.203x+0.147$$

where *x* is the capsule length and *y* is the precision.

The fitting-curve can be further used for predicting confidence assessment: given a capsule length, its prediction confidence can be estimated using the above equation.

### Comparison with previous predictors

For comparing with other predictors, the public benchmark GT320 was used. Following the previous studies, a five-fold cross validation was conducted. This GT320 is an imbalanced dataset, but for objective evaluation, we did not sample any balanced data from training or testing, as done in previous studies. Table 5 shows that the proposed DeepICN outperformed all the previous methods by a significant margin.

**Table 5:** Performance comparison with previous predictors using the GT320 benchmark.

Methods	MCC
**Our Approach**	**0.45**
Zhu *et al*.^20^	0.38
Hu’s SVM	0.18
SNNS	0.17
GTSVM	0.12
WEKA-logistic regression	0.12
WEKA-naïve Bayes	0.11

The results of WEKA, SNNS were obtained from the paper^11^, the result of GTSVM was obtained from the paper^12^ and result of Hu’s SVM was from the paper^19^. Zhu *et al*.^20^ is the previous best predictor.

### Extension of DeepICN for classic and inverse gamma-turn prediction

**Figure 3:**
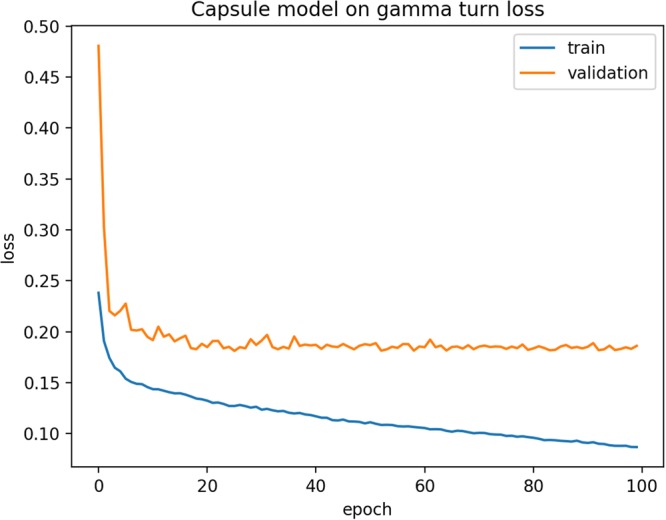
Training loss and validation loss curves of DeepICN for classic and inverse gamma-turn.

Many previous gamma-turn predictors only predict whether a turn is gamma-turn or not. Here, we also extended our DeepICN model for classic and inverse gamma-turn prediction. The experiment dataset is CullPDB, and inverse and classic labels were assigned using PROMOTIF^9^. The same DeepICN (as described in Methods) was applied except the last turn capsule layer now has three capsules to predict non-turn, inverse turn or classic turn as a three-class classification problem. The performance metric Q3 is used which is the accuracy of correct prediction for each class. The prediction results are shown in Table 6. Different numbers of proteins were used to build the training set. The validation and test set contain 500 proteins each. The CullPDB dataset contains 10,007 proteins which have 1383 classic turns, 17,800 inverse turns, and 2,439,018 non-turns in total. This is a very imbalanced dataset. In this experiment, the balanced training set, validation set, and test set were generated as follows: The inverse turn samples were randomly drawn as many as classic turn sample size. For the non-turn samples, they were randomly drawn twice as many as classic turn sample size, i.e. the sum of inverse turn samples and classic turn samples. The training loss and validation loss curves are shown in Fig. 3. From the loss curve, it shows that after about 75 epochs, the model learning process was converging. Since the model hyper-parameters had been explored in the earlier experiments, during this experiment, we adopted similar values, i.e., the window size was chosen 17 amino acids, the filter size is 256, the convolution kernel size was chosen 3, the dynamic routing was chosen 3 iterations and the dropout ratio was 0.3.

**Table 6:** Non-turn, inverse and classic turn prediction results.

Training size	Test average Q3	Time (hr)	P-value
5000	0.6839 (±0.0053)	0.25 (±0.20)	—
6000	0.6783 (±0.0076)	0.38 (±0.22)	0.2706
7000	0.6864 (±0.0124)	0.34 (±0.16)	0.3057

### Visualization of the features learnt by capsules

**Figure 4:**
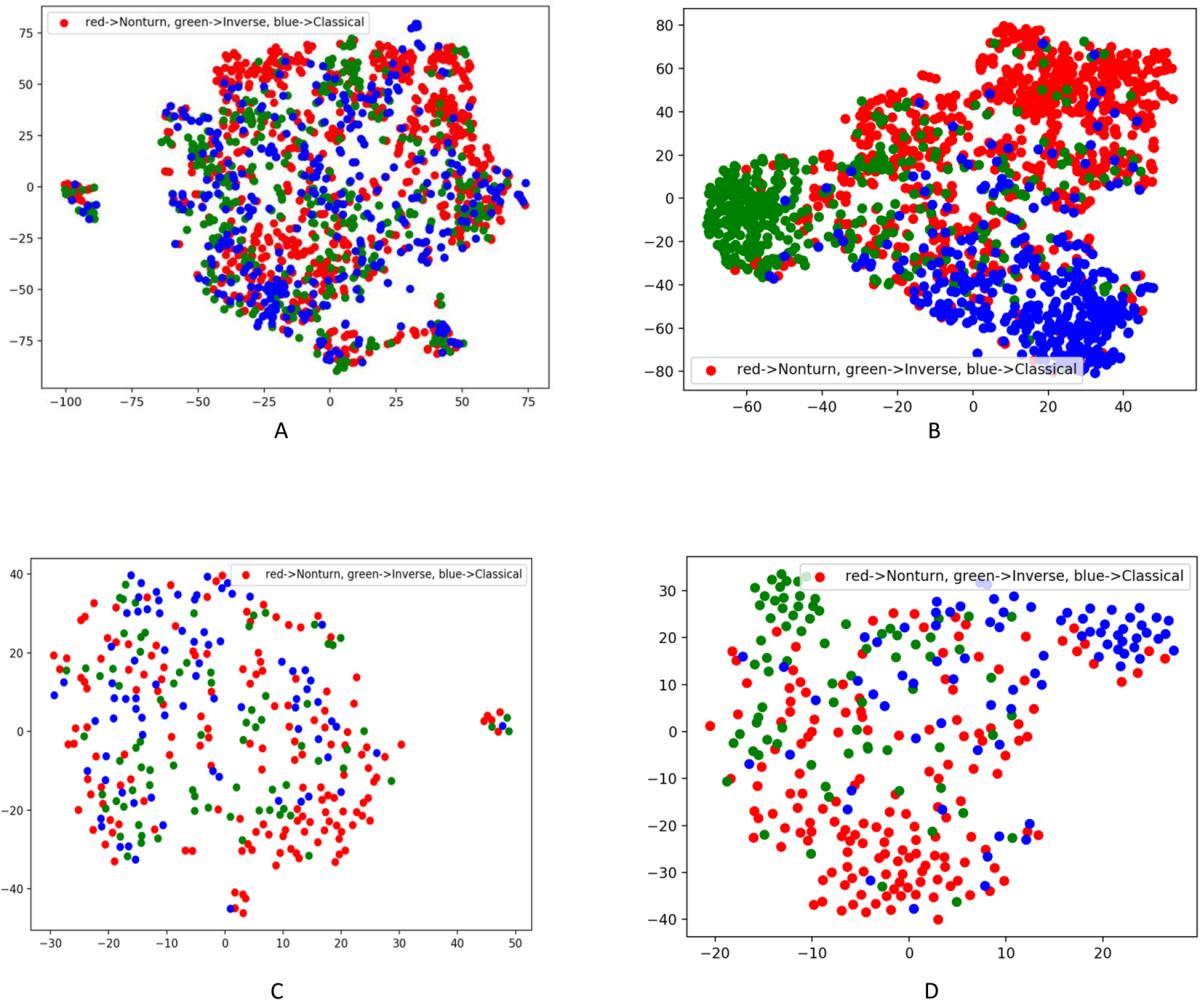
t-SNE plots of DeepICN features. (**A,B**) Are plots of the input features and the capsule features, respectively for training dataset (3000 proteins with 1516 turn samples). (**C,D**) Are plots of the input features and the capsule features, respectively for the test dataset (500 proteins with 312 turn samples). Red dots represent non-turns, green dots represent inverse turns and blue dots represent classic turns.

In order to verify whether the high-level features learnt/extracted from the input data have the prediction power and are generalizable, t-SNE^33^ was applied to visualize the input features and the capsule features for both the training data and the test data. Figure 4(A) shows the t-SNE plot of the input features from the training data before the training. The input data has 45 features (i.e. 45 dimensions), and t-SNE can project 45 dimensions onto two principal dimensions and visualize it. There was no clear cluster in the training data. Figure 4(B) shows the t-SNE plot of the capsule features from the training data. The turn capsule contains 16 dimensions, and the t-SNE can similarly project the capsule features to two major principal features and visualize it. The clusters were obviously formed after the training. Figure 4(C,D) show the t-SNE plots for the input features and the capsule features of the test data. There was no clear cluster for the input features in the test data either. The capsule features still tend to be clustered together in the test data, although to less extent than the training data.

**Figure 5:**
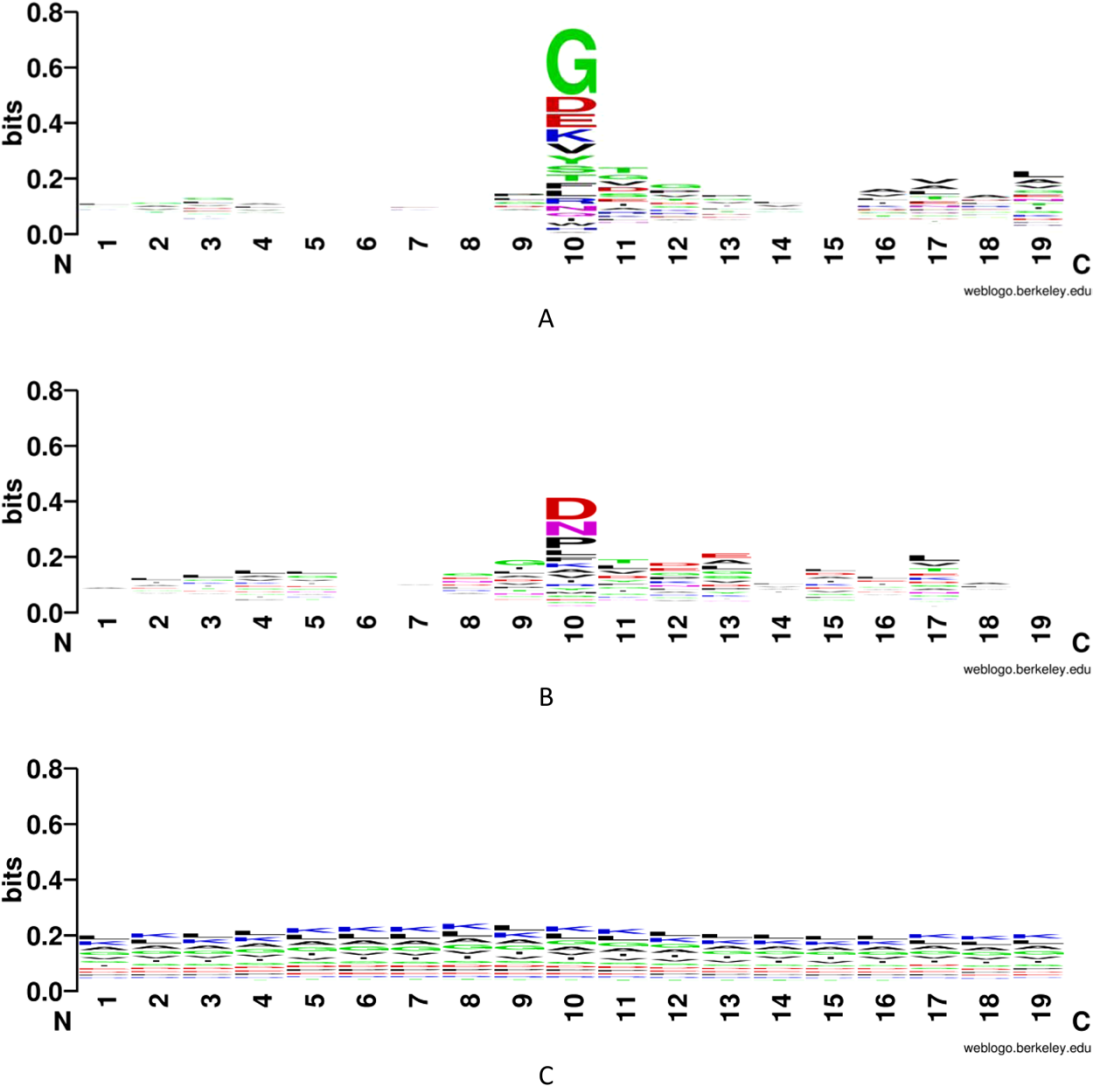
Weblogo of sequence types. (**A**) Classic turn; (**B**) inverse turn; and (**C**) non-turn.

Figure 5(A) shows the classic turn Weblogo^34^ and Fig. 5(B) shows the inverse turn Weblogo, with a length of 19 amino acids. The middle three amino acids (at 9, 10, 11) represent the key positions of an inverse turn or classic turn. Eight amino acids are extended to each side of these three amino acids. We randomly selected 300 inverse turn, classic turn or non-turn fragments from the training set to plot Weblog. In the two plots, the y axis has the same height of 0.8 bits. Both types of turns have some visible features and the classic turn Weblogo contains more information content than the inverse turn.

### Ablation study

To compare the performance between DeepICN and CNN, we designed a set of experiments by removing different components in the DeepICN or replacing them with CNN. In particular, we tested the performance of the proposed models without the capsule component, replacing the capsule component with CNN, or replacing inception component with CNN. Each ablation experiment was performed using the same allocation of the data (3000 proteins for training, 500 proteins for validation, and 500 for test) and the same parameter setting: dropout ratio 0.5 and window size 17. From the ablation test result presented in Table 7, we found that the capsule component is the most effective component in our network, since the performance dropped significantly when removing or replacing the capsule component. The inception component also acts as an important component as it can more effectively extract feature maps for capsule components than CNN.

**Table 7:** Ablation test.

Model	MCC
Replace inception component with CNN	0.4544 (±0.0106)
Replace capsule component with CNN	0.4485 (±0.0056)
Without capsule component	0.4551 (±0.0059)
**Proposed Design**	**0.4752 (±0.0111)**

### Additional test using CullPDB as training and GT320 as testing

To further validate the prediction performance, we designed a cross-dataset experiment between two datasets, i.e., using CullPDB as the training set and the GT320 as the testing set. The CullPDB after preprocessing (see the “experimental data set” section for details) contains 10,007 proteins. To perform a strict and objective test, we applied CD-Hit^32^ with 30% sequence similarity cutoff between CullPDB and GT320 to remove similar sequences from CullPDB, leaving 9,837 proteins in CullPDB. We randomly selected 800 proteins for training and 200 protein for validation in CullPDB. The training set contained 188,436 non-turn samples and 1505 turn samples; the validation set contained 47,182 non-turn samples and 369 turn samples; the test set contained 79,646 non-turn samples and 892 turn samples. The training, validation and testing were carried out using the unbalanced data in the same fashion. The average training time was about 5–6 hours. The parameter settings were: dynamic routing three times, window size 17 and dropout rate 0.5. The average training MCC was 0.5552 and the average validation MCC was 0.5149. The test MCC for GT320 was 0.4571, which is similar to our result in Table 5 and confirmed the robustness of our model.

## Conclusion and Discussion

In this work, the newly proposed deep-learning framework, CapsuleNet, was applied to protein gamma-turn prediction. Instead of applying capsule network directly, a new model called inception capsule network was proposed and has shown improved performance comparing to previous predictors. This work has several innovations.

First of all, this work is the first application of deep neural networks to protein gamma-turn prediction. Compared to previous traditional machine-learning methods for protein gamma-turn prediction, this work uses a more sophisticated, yet efficient, deep-learning architecture, which outperforms previous methods. A software tool has been developed and it will provide the research community a powerful deep-learning prediction tool for gamma-turn prediction. The ablation test was performed, and the importance of capsule component was verified.

Second, this work is the first application of CapsuleNet to protein structure-related prediction, as CapsuleNet was just published in 2017. Here, we proposed DeepICN for protein gamma-turn prediction and explored some unique characters of capsules. To explore the capsule length, we designed an experiment of grouping each capsule length into several bins and discovered the relationship between prediction precision and capsule length. A nonlinear curve can be applied to fit the data and further used for estimating the prediction confidence. In addition, the network was extended to inverse turn and classical turn prediction. The inverse turn capsule and classical turn capsule were further explored by showing the t-SNE visualization of the learnt capsule features. Some interesting motifs were visualized by Weblogo.

Third, new features have been explored and applied to gamma-turn prediction. The features used for network training, namely HHBlits profiles and predicted shape strings, contain high information content making deep learning very effective. The HHBlits profiles provide evolutionary information while shape strings provide complementary structural information for effectively predicting gamma turns.

Last but not least, previous gamma-turn resources are very limited and outdated. Previous servers are not maintained, and no downloadable executable of gamma-turn is available. We will provide a new free tool utilizing deep learning and state-of-the-art CapsuleNet for researchers.

While we have obtained encouraging results, we believe the performance can be further improved by using different deep neural network models and additional features. For instance, the chemical shift information has been used in many protein structure studies^35–37^. We consider using such information to further improve the model performance in the future work.

## Methods

### Problem formulation

A protein gamma-turn prediction is a binary classification problem, which can be formulated as followed: given a primary sequence of a protein, a sliding window of ***k*** residues were used to predict the central residue turn or non-turn. For example, if ***k*** is 17, then each protein is subsequently sliced into fragments of 17 amino acids with a sliding window. The reason of using sliding window is that gamma turn is very sparse in a protein sequence, which is ineffective to predict if using the whole sequence like deep learning prediction for protein secondary structures^21,22^.

To make accurate prediction, it is important to provide useful input features to machine-learning methods. We carefully designed a feature matrix corresponding to the primary amino acid sequence of a protein, which consists of a rich set of information derived from individual amino acid, as well as the context of the protein sequence. Specifically, the feature matrix is a composition of HHBlits profile^30^ and predicted protein shape string using Frag1D^31^.

The first set of features comes from the protein profiles generated using HHBlits^30^. In our experiments, HHBlits used the database uniprot20_2013_03, which was downloaded from http://wwwuser.gwdg.de/compbiol/data/hhsuite/databases/hhsuite_dbs/. A HHBlits profile can reflect the evolutionary information of the protein sequence based on a search of the given protein sequence against a sequence database. The profile values were scaled by the sigmoid function into the range (0, 1). Each amino acid in the protein sequence is represented as a vector of 31 real numbers, of which 30 from HHM profile values and 1 *NoSeq* label (representing a gap) in the last column. The HHBlits profile corresponds to amino acids and some transition probabilities, i.e., A, C, D, E, F, G, H, I, K, L, M, N, P, Q, R, S, T, V, W, Y, M- > M, M- > I, M- > D, I- > M, I- > I, D- > M, D- > D, Neff, Neff_I, and Neff_D.

The second set of features, predicted shape string, comes from Frag1D^31^. For each protein sequence, Frag1D can generate predicted protein 1D structure features: classical three-state secondary structures, and three- and eight-state shape strings. Classical three-state secondary structures and three-state shape string labels both contain H (helix), S (sheet), and R (random loop), but they are based on different methods so that they have small differences. In this experiment, we used all the features from Frag1D. Eight-state shape string labels contain R (polyproline type alpha structure), S (beta sheet), U/V (bridging regions), A (alpha helices), K (3_10_ helices), G (almost entirely glycine), and T (turns). The classical prediction of three-state protein secondary structures has been used as an important feature for protein structure prediction, but it does not carry further structural information for the loop regions, which account for an average of 40% of all residues in proteins. Ison *et al*.^38^ proposed Shape Strings, which give a 1D string of symbols representing the distribution of protein backbone psi-phi torsion angles. The shape strings include the conformations of residues in regular secondary structure elements; in particular, shape ‘A’ corresponds to alpha helix and shape ‘S’ corresponds to beta strand. Besides, shape strings classify the random loop regions into several states that contain much more conformational information, which we found particularly useful for gamma-turn prediction problem. For the Frag1D prediction result, each amino acid in the protein sequence is represented as a vector of 15 numbers, of which 3 from the classical three-state secondary structures, 3 from the three-state shape strings, 8 from the eight-state shape strings and 1 *NoSeq* label in the last column. The predicted classical three-state secondary structure feature is represented as one-hot encoding as followed: helix: (1, 0, 0), strand: (0, 1, 0), and loop: (0, 0, 1). The same rule applies to three- and eight-state shape string features. In this work, we also tried the traditional eight-state protein secondary structures. However, the prediction result was not as good as the one from the eight-state shape strings. This is probably because the traditional eight-state secondary structures contain much less structural information for the gamma-turn prediction problem.

### Model design

**Figure 6:**
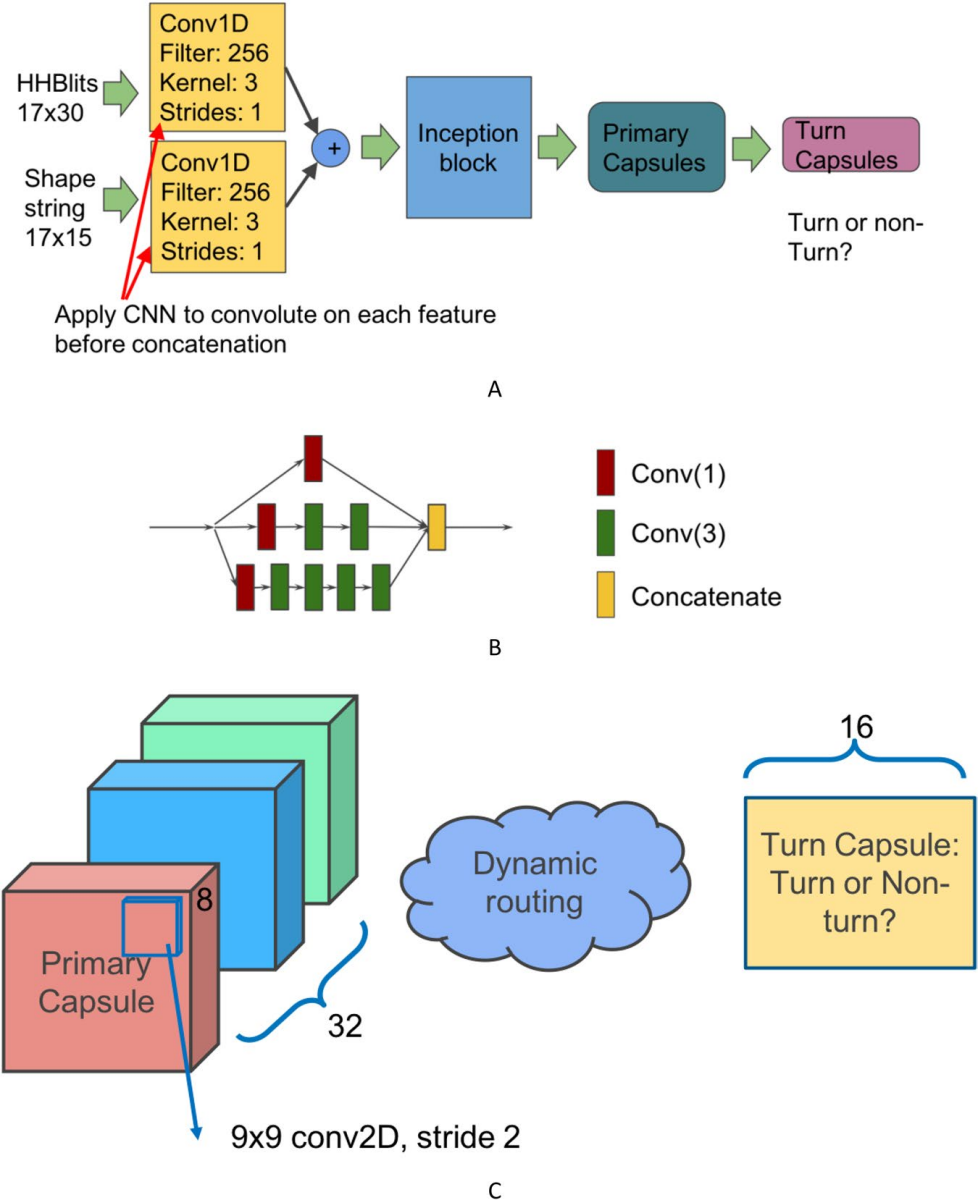
(**A**) DeepICN design. The input features are HHBlits profiles (17-by-30 2D array) and predicted shapes string using Frag1D (17-by-15 2D array). Each feature is convolved by a convolutional layer. Both convolved features then get concatenated. An inception block is followed to extract low-to-medium features. A primary capsule layer then extracts higher level features. The final turn capsule layer makes predictions. (**B**) An inception block. Inside this inception block: Red square Conv(1) stands for convolution operation with kernel size 1. Green square Conv(3) stands for convolution operation with kernel size 3. Yellow square stands for feature map concatenation. (**C**) Zoom-in between primary capsules and turn capsules. The primary capsule layer contains 32 channels of convolutional 8D capsules. The final layer turn capsule has two 16D capsules to represent two states of the predicted labels: gamma-turn or non-gamma-turn. The computation between those two layers is dynamic routing.

In this section, a new deep inception capsule network (DeepICN) is presented. Figure 6A shows the model design. The input features for DeepICN are HHBlits profiles and predicted shape strings. Since the distributions of HHBlits profiles and predicted shape strings are different, we applied convolutional filters separately on the two features, then concatenated them. The CNN is used to generate the convolved features. We first applied CNN to extract local low-level features from protein profiles and predicted shape strings features. This CNN layer will extract local features similar to a CNN used to extract “edge” features of objects in an image^39^.

After the convoluted feature concatenation, the merged features are fed into the inception module (see Fig. 6B for details). The inception network was then applied to extract low-to-intermediate features for CapsuleNet. CapsuleNet was originally used for digital image classification^26^ and the primary capsule layers were placed after a convolutional layer. Their network design worked well for digital image recognition with the image dimension 28-by-28. Considering the complex features of protein HHblits profile and shape strings, it is reasonable to apply a deeper network to extract local-to-medium level features so that CapsuleNet can work well on top of those features and extract high-level features for gamma-turn classification. The purpose of setting up an inception block right after CNN is to extract intermediate-level features.

Each convolution layer, such as ‘Conv (3)’ in Fig. 6B, consists of four operations in the sequential order: (1) a one-dimensional convolution operation using the kernel size of three; (2) the batch normalization technique^40^ for speeding up the training process and acting as a regularizer; (3) the activation operation, ReLU^41^; and (4) the dropout operation^42^ to reduce the overfitting effects by randomly dropping neurons during the deep network training process so that the network can avoid co-adapting.

The capsule layers are placed after the inception module to extract high-level features and explore the spatial relationships among the local features that are extracted in the above-mentioned layers. The primary capsule layer (see Fig. 6C) is a convolutional capsule layer as described in the paper^26^. It contains 32 channels of convolutional 8D capsules, with a 9 × 9 kernel and a stride of 2. The final layer (turn capsule) has two 16D capsules to represent two states of the predicted label: gamma-turn or non-gamma-turn. The weights between primary capsules and turn capsules are determined by the iterative dynamic routing algorithm^26^. The squashing activation function^26^ was applied in the computation between the primary capsule layer and the turn capsule layer as follows:

$${v}_{j}=\frac{\Vert {s}_{j}\Vert }{1+{\Vert {s}_{j}\Vert }^{2}}\frac{{s}_{j}}{\Vert {s}_{j}\Vert }$$

where *v*_*j*_ is the scaled vector output of capsule *j* and *s*_*j*_ is its total output.

The dynamic routing algorithm^26^ is as follows:

**Algorithm 1:**
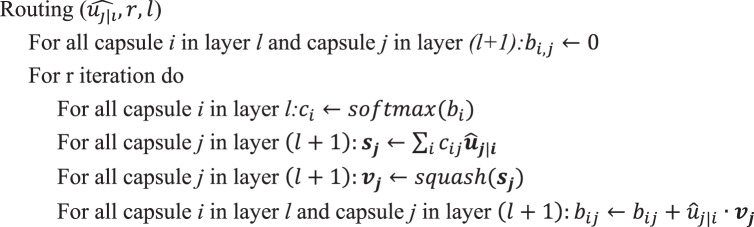
Routing Algorithm.

The evaluation matric for gamma-turn prediction is Matthew Correlation Coefficient (MCC), which is more commonly used than accuracy since accuracy only considers the true positives and false positives, not the true negatives and false negatives. Another reason is that the gamma-turn dataset is very imbalanced. MCC can evaluate how well the classifier performs on both positive and negative labels. MCC can be calculated from the confusion matrix as follows:

$$MCC=\frac{TP\,\ast \,TN-FP\,\ast \,FN}{\sqrt{(TP+FP)(TP+FN)(TN+FP)(TN+FN)}}$$

where TP is the number of true positives, TN is the number of true negatives, FP is the number of false positives and FN is the number of false negatives.

### Model training

DeepICN was implemented, trained, and tested using TensorFlow and Keras. Different sets of hyper-parameters (dynamic routing iteration times, training data sample size, convolution kernel size, and sliding window size) of DeepICN were explored. An early stopping strategy was used when training the models: if the validation loss did not reduce in 10 epochs, the training process was stopped. The Adam optimizer was used to dynamically change the learning rate during model training. All the experiments were performed on an Alienware Area-51 desktop equipped with a Nvidia Titan X GPU (11 GB graphic memory).
